# Evaluation of six clinical prognostic scores in NSCLC patients undergoing first line chemoimmunotherapy

**DOI:** 10.3389/fimmu.2026.1695859

**Published:** 2026-02-17

**Authors:** Jiaqi Sun, Dan Li, Jiayin Liu, Lan Wang, Long Wang, Jing Han, Xue Zhang, Xinliang Zhou, Li Feng, Zhisong Fan, Jing Zuo, Yudong Wang

**Affiliations:** 1 Department of Medical Oncology, The Fourth Hospital of Hebei Medical University, Shijiazhuang, Hebei, China; 2 Department of Respiratory, Beijing Genertec Aerospace Hospital, Beijing, China

**Keywords:** chemoimmunotherapy, clinical prognostic scores, efficacy, first-line anti-tumor treatment, NSCLC, prognosis

## Abstract

**Background:**

The study aimed to evaluate the effectiveness of six prognostic scores for predicting the outcomes to first-line chemoimmunotherapy (CIT) in non-small cell lung cancer (NSCLC) patients.

**Materials and methods:**

NSCLC patients receiving first-line CIT were included. The prognostic scores evaluated were RMH, MDACC, MDACC+NLR, MDA-ICI, LIPI, and GRIm. Survival curves were generated using the Kaplan–Meier method, and univariate and multivariate analyses were conducted via the Cox proportional hazards regression model. The C−index and time−dependent AUC were calculated to comprehensively quantify and compare the predictive performance of each system. The Log−rank test and False Discovery Rate (FDR) correction was employed to compare survival outcomes across different risk groups defined by the six prognostic scoring systems.

**Results:**

A cohort of 298 NSCLC patients was analyzed. The median overall survival (mOS) of patients receiving first-line CIT was 36.5 months (95%CI: NE-NE), and the median progression-free survival (mPFS) was 14.5 months (95%CI: 11.9-17.1). Multivariate analysis showed that bone metastasis (*P* = 0.042), and more than two metastatic sites (*P* = 0.031) as independent predictors of poor OS. In quantitative performance comparison, RMH achieved the highest C-indices for both OS (0.672, 95%CI: 0.531-0.813) and PFS (0.652, 0.564-0.737); MDACC also performed well, with C-indices for OS (0.651, 0.564-0.737) and PFS (0.615, 0.554-0.738). Time-dependent AUC analysis showed that MDA-ICI attained the highest 1-year OS and PFS AUC (0.630 and 0.592), followed by the MDACC+NLR (0.600 and 0.571). Based on log-rank testing and following FDR correction, only the MDACC maintained a statistically significant association with OS (high-risk 14.0 vs. intermediate-risk 34.6 vs. low-risk NR months; P = 0.003, Q = 0.036). For PFS, the MDACC+NLR score showed a marginal significance after FDR correction (Q = 0.054).

**Conclusions:**

The RMH, MDACC, and MDACC+NLR scoring systems all demonstrate prognostic utility in the NSCLC patients treated with first-line CIT, and the optimal choice among them may depend on the specific clinical context and the outcome metric of primary interest.

## 1 Introduction

Lung cancer was the most common cancer in China in 2022 (1,060,600 cases) for both sexes combined, the leading cause of cancer death was lung cancer (733,300 deaths) (1). About 80–85% of all lung malignancies, including adenocarcinoma, squamous cell carcinoma, and other histologic subtypes, are classified as non-small cell lung cancer (NSCLC) (2). Most NSCLC patients are often not diagnosed until the disease has progressed to metastatic or locally advanced stages due to the lack of early clinical symptoms. In individuals who undergo surgical resection for resectable NSCLC, the possibility of postoperative metastatic recurrence cannot be disregarded (3). Common sites of metastasis include the brain, bones, and lungs, among others, leading to a significant decline in both patient quality of life and survival rates. Therefore, improving long-term survival outcomes necessitates early detection and accurate prognostic assessment of patients.

In recent years, immunotherapy, particularly immune checkpoint inhibitors (ICIs), has revolutionized the treatment of anticancer therapies. When combined with chemotherapy, ICIs have shown significant efficacy in slowing disease progression in locally advanced or metastatic NSCLC. As a result, chemoimmunotherapy (CIT) has emerged as the preferred treatment approach for advanced driver-negative NSCLC (4). However, it is important to note that not all patients benefit equally from CIT. Therefore, further research is needed to identify and screen patients who are more likely to respond favorably to CIT. This will enable the development of personalized treatment plans, maximize therapeutic benefits, and minimize the risk of overutilization and potential adverse effects.

Currently, there is a lack of reliable biomarkers that can accurately predict the efficacy and prognosis of CIT in NSCLC patients. However, several composite scoring systems have been developed and validated in various cohorts of advanced NSCLC patients to assist in predicting outcomes. These scoring systems include the Royal Marsden Hospital score (RMH), MD Anderson Cancer Center score (MDACC), MDACC combined with Neutrophil-to-Lymphocyte Ratio (NLR), MD Anderson Immune Checkpoint Inhibitor (MDA-ICI) score, Lung Immune Prognostic Index (LIPI), and Gustave Roussy Immune Score (GRIm) (5–10). These scoring systems serve as valuable tools in identifying patients who may benefit from novel treatments and in assessing the likelihood of survival.

However, it remains unclear how effectively these existing scoring systems can be utilized to evaluate the prognosis and effectiveness of first-line CIT in NSCLC patients. Further research and validation studies are needed to determine the utility of these scoring schemes specifically in the context of first-line CIT for NSCLC patients.

In this study, our goal was to investigate the clinicopathological characteristics and survival outcomes of NSCLC patients who received CIT combinations. Additionally, we aimed to assess the utility of six prognostic scoring systems in evaluating the effectiveness and prognosis of first-line CIT in NSCLC. Our objective was to identify the most reliable prognostic scoring method that could guide the selection of patients who would benefit most from first-line CIT, thereby optimizing personalized treatment strategies.

## 2 Materials and methods

### 2.1 Patients

In this study, we conducted a retrospective analysis by reviewing the electronic medical records of NSCLC patients who received first-line CIT combination regimens at the Fourth Hospital of Hebei Medical University. The data collection period spanned from 2019 to 2023. Patients were enrolled if they met all of the following criteria: (1) histologically or cytologically diagnosed with primary NSCLC and clinically diagnosed as inoperable (including patient refusal of surgery) or metastatic; (2) age ≥18 years; (3) no contraindications to ICIs or chemotherapy based on laboratory tests and examinations; (4) having received at least two cycles of first-line chemoimmunotherapy (CIT) with evaluable efficacy; (5) availability of imaging evaluations (e.g., CT, MRI, bone scan) for tumor response assessment; (6) complete clinicopathological data, genetic testing results, baseline peripheral blood test results, treatment records, and available follow-up data; (7) driver gene-negative status (no mutations in EGFR, ALK, ROS1, c-MET, BRAF, RET, HER2, or NTRK), primarily confirmed by next-generation sequencing (NGS, with no restrictions on gene panel size). Patients were excluded based on any of the following: (1) history of a second primary malignancy; (2) concurrent chronic or acute infection, severe autoimmune disease, or ongoing immunosuppressive therapy; (3) presence of severe cardiovascular or cerebrovascular diseases, or dysfunction of major organs such as the liver or kidneys; (4) inability to cooperate during follow-up, or having complex clinical conditions resulting in incomplete data. This study was approved by the Ethics Committee of the Fourth Hospital of Hebei Medical University (approval number: 2022KY435).

### 2.2 Data

We collected data including clinicopathological features, peripheral blood biomarkers, diagnostic and treatment procedures, and survival status of all patients. The following demographics were recorded for analysis: sex; age; histologic subtype of NSCLC; Eastern Cooperative Oncology Group Performance Status (ECOG PS); smoking history; clinical stage; PD-L1 status when available; sites of metastasis; radiotherapy history; immunotherapy modality; efficacy; date of progression, and survival (death or last follow-up). Baseline peripheral blood indicators, defined as the most recent blood samples collected within 1 week prior to the initiation of CIT treatment, were systematically documented in the study database for all enrolled patients. These indicators included white blood cell (WBC) count, absolute neutrophil count (ANC), absolute lymphocyte count (ALC), platelet count, lactate dehydrogenase (LDH) level, and serum albumin level. During the first-line treatment period, all occurring treatment-related adverse events (TRAEs) were systematically collected and graded according to the Common Terminology Criteria for Adverse Events (CTCAE) version 4.0 (Refer to **Supplementary Table 1** for details).

PD-L1 expression was assessed using the 22C3 pharmDx assay on the Dako Autostainer Link 48 immunohistochemistry platform. The tumor proportion score (TPS) was used as the evaluation metric, with results categorized as follows: negative (TPS < 1%), low expression (TPS 1%–49%), and high expression (TPS ≥ 50%).

### 2.3 Therapeutic regimen

All enrolled patients received first-line CIT combination regimens. For patients with squamous cell carcinoma, chemotherapy regimens included taxanes combined with platinum-based agents, single-agent taxanes, and gemcitabine combined with platinum-based agents. For patients with non-squamous carcinoma, chemotherapy regimens included pemetrexed combined with platinum-based agents, taxanes combined with platinum-based agents, single-agent pemetrexed, and single-agent taxanes. Immunotherapeutic agents included nivolumab, pembrolizumab, durvalumab, tislelizumab, camrelizumab, sintilimab and toripalimab. During the treatment course, chemotherapy agents combined with immunotherapeutic agents were continued for 4–6 cycles, followed by maintenance therapy with immunotherapeutic agents until disease progression or the occurrence of unacceptable toxicity.

### 2.4 Survival assessments

Tumor response was evaluated through computed tomography every two months according to response evaluation criteria in solid tumors criteria (RECIST) V1.1. The overall survival (OS) was measured the time from the start of first-line treatment until death or final follow-up; the progression-free survival (PFS) was measured the time from the start of first-line treatment until disease progression or death. If the patient had not experienced disease progression or death at the time of data cutoff, the date of last follow-up was used as the cutoff value.

### 2.5 Comparison of six prognostic scoring systems

We calculated RMH, MDACC, MDACC + NLR, MDA-ICI, LIPI, and GRIm prognostic scores for each patient. Patients were categorized into different prognostic risk groups according to the established cut-offs for each score. See **Table 1** for full details of each scores.

**Table 1:** Components and risk categorization of prognostic scoring systems.

Prognostic scoring system	Components	Point	Risk categories
RMH	LDH^1^ > ULN^2^	1	Low risk: 0-1 High risk: 2-3
	ALB^3^ < 35g/L	1	
	> 2 metastatic sites	1	
MDACC	> 2 metastatic sites	1	Low risk: 0-1 Intermediate risk: 2 High risk: 3-5
	ECOG PS^4^ ≥ 1	1	
	LDH > ULN	1	
	ALB < 35g/L	1	
	Gastrointestinal tumour^5^	0	
MDACC + NLR	> 2 metastatic sites	1	Low risk: 0-1 High risk: > 1
	ECOG PS ≥ 1	1	
	LDH > ULN	1	
	ALB < 35g/L	1	
	NLR^6^ >6	1	
	Gastrointestinal tumor	0	
MDA-ICI	Age >52 years old	1	Low risk: 0-2 Intermediate risk: 3 Intermediate-high risk: 4 High risk: 5-7
	Liver metastasis	1	
	ECOG PS > 1	1	
	ANC > 4.9×10^9^/L	1	
	ALC < 1.8×10^9^/L	1	
	PLT > 300 counts	1	
	LDH > 75%ULN^7^	1	
LIPI	dNLR^8^ > 3 counts	1	Low risk: 0 Intermediate risk: 1 High risk: 2
	LDH > ULN	1	
GRIm	NLR >6	1	Low risk: 0-1 High risk: 2-3
	LDH > ULN	1	
	ALB < 35g/L	1	

^1^LDH, lactate dehydrogenase. ^2^ULN, upper limit of normal, in this study, ULN was set at 245U/L. ^3^ALB, albumin. ^4^ECOG PS, Eastern Cooperative Oncology Group performance status. ^5^In this study, the score item of “gastrointestinal tumor” in all patients was recorded as 0. ^6^NLR, neutrophil-to-lymphocyte ratio. ^7^75%ULN, in this study, 75%ULN was set at 183U/L. ^8^dNLR, derived neutrophil-to-lymphocyte ratio.

We established an assessment framework centered on discriminative ability. Specifically, the C-index and the time-dependent AUC were employed as primary metrics to evaluate the models’ discriminatory performance. Complementarily, the log-rank test (with FDR-adjusted Q-values) was employed to formally assess the statistical significance and clinical validity of the survival differences between the predefined risk strata generated by each model.

### 2.6 Statistical analysis

The software SPSS version 21.0 and R (version 4.4.2; R Foundation for Statistical Computing, Vienna, Austria) was used to analyze the data. Values and percentages were used to express the count data, and the chi-square test was used to statistically assess the comparisons. The Kaplan-Meier survival curve method was used to compare the survival times of the groups, and the Log-rank test was used to look for significant differences. The COX regression model was utilized to examine prognostic factors and determine adjusted hazard ratios (HR) along with their 95% confidence intervals (CIs). Variables with *P* < 0.1 in univariate analysis were entered into multivariable Cox regression models to identify independent prognostic factors. Before conducting the multiple regression analysis, we used the *car* package to calculate the variance inflation factor (VIF) in order to assess the multicollinearity among the independent variables. A VIF value of less than 5 was set as the threshold to indicate the absence of severe multicollinearity. Apart from that, the proportional hazards (PH) assumption for covariates was assessed using Schoenfeld residuals with the *cox.zph* function in *survival* package. A significant p-value (<0.05) indicated a violation of the PH assumption. The *survcomp* package was used to calculate the C-index, while the *timeROC* package was utilized to construct time-dependent ROC curves and compute the AUC at different time points. Data visualization was carried out using the *ggplot2* package. A statistically significant difference was defined as a test level of P < 0.05. Given the multiple comparisons performed by evaluating six different scoring systems on the same patient cohort, the resulting P values were adjusted for multiple testing using the Benjamini-Hochberg (BH) method to control the False Discovery Rate (FDR). Statistical significance after correction was defined as an FDR-adjusted Q < 0.05.

## 3 Results

### 3.1 Patient characteristics

A total of 298 consecutive patients with unresectable/metastatic NSCLC who received first-line chemoimmunotherapy between August 2019 and July 2023 were included. The median age of the patients was 64.0 (range, 32.0-86.0) years. Most of the patients were male (n=253, 84.9%) and former or current smokers (n=194, 65.1%). The ECOG score was 0 in 110 cases (36.9%), 1 in 152 cases (51.0%), and ≥2 in 36 cases (12.1%). 49.3% of the patients were diagnosed as having squamous cell carcinoma. 138 patients (46.3%) with no distant organ metastasis at the initial diagnosis, 76 patients (25.5%) with single distant organ metastasis, and 84 patients (28.2%) with two or more multiple distant metastases; 21.5% of patients had baseline bone metastasis, and 9.1% of patients had baseline liver metastasis. The other clinical characteristics and laboratory parameters are shown in **Table 2**.

**Table 2:** Baseline patient and tumor characteristics.

Parameters	N = 298 (%)
Sex	
Female	45 (15.1)
Male	253 (84.9)
Age	
≥65	133 (44.6)
<65	165 (55.4)
Median (range)	64 (32-86)
ECOG PS^1^	
0	110 (36.9)
1	152 (51.0)
≥2	36 (12.1)
Pathology	
Squamous cell carcinoma	147 (49.3)
Adenocarcinoma	141 (47.3)
Others^2^	10 (3.4)
Smoking status	
Yes	194 (65.1)
No	104 (34.9)
Previous chronic history	
Yes	188 (63.1)
No	110 (36.9)
Metastatic site	
Brain	43 (14.4)
Bone	64 (21.5)
Lung	57 (19.1)
Pleural	49 (16.4)
Liver	27 (9.1)
Adrenal glands	21 (7.0)
Number of metastasis organs	
0	138 (46.3)
1	76 (25.5)
2	49 (16.4)
>2	35 (11.7)
PD-L1 status	
<1%	33 (11.1)
1%-49%	64 (21.5)
≥50%	41 (13.7)
Unknown	160 (53.7)
First-line clinical stage	
II	14 (4.7)
III	117 (39.3)
IV^3^	167 (56.0)
Radiotherapy history	
Yes	65 (21.8)
No	233 (78.2)

^1^ECOG PS, Eastern Cooperative Oncology Group performance status. ^2^”Others” included adenosquamous carcinoma (n=7), adenocarcinoma with sarcomatosis (n=2), and undifferentiated carcinoma (n=1). ^3^”Stage IV” included metastatic NSCLC (n=160) and postoperative recurrence (n=7).

### 3.2 Efficacy and survival

**Figure 1:**
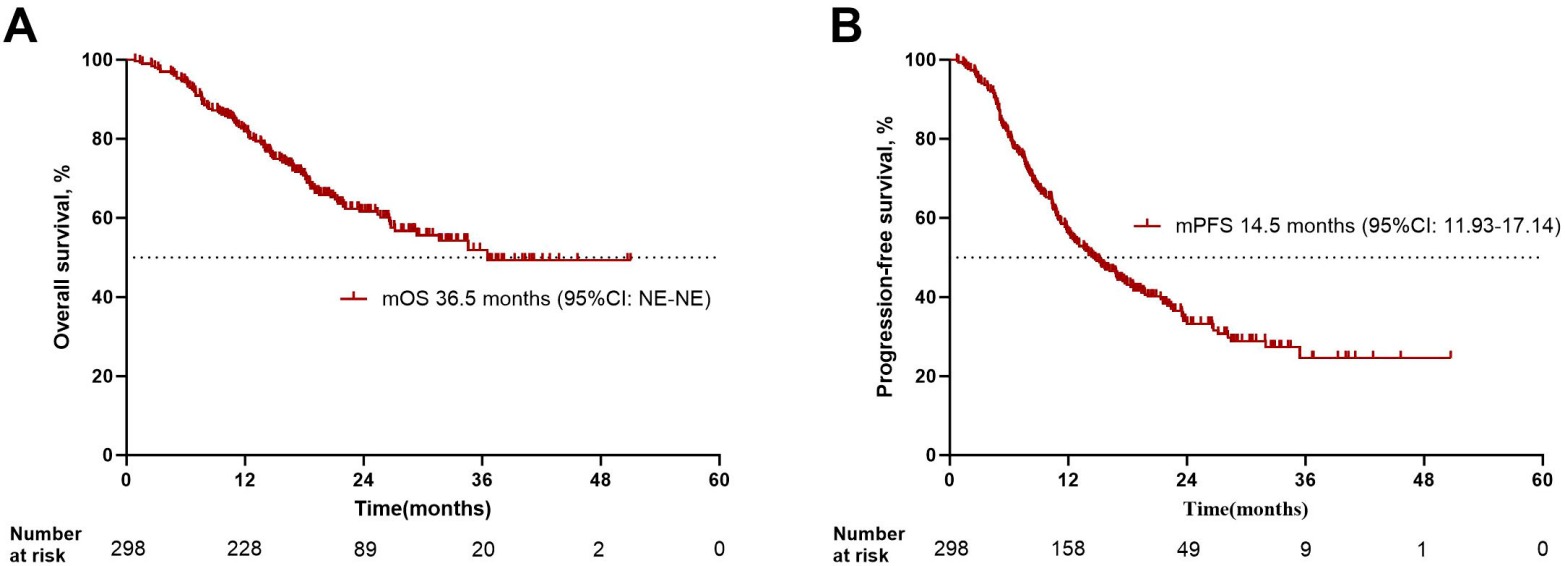
Overall survival and progression-free survival in NSCLC patients treated on first-line CIT. **(A)** Kaplan-Meier survival analysis for overall survival in all patients treated on first-line CIT. **(B)** Kaplan-Meier survival analysis for progression-free survival in all patients treated on first-line CIT. The median duration of follow-up was 23.7 (95% CI: 21.5-25.8) months.

All patients in this study received a first-line CIT combination regimen, and RECIST version 1.1 was applied to evaluate the efficacy of 298 NSCLC patients. The median duration of follow-up was 23.7 (95% CI: 21.5-25.8) months. The results showed that: there was 1 case of CR (0.3%), 138 cases of PR (46.3%), 132 cases of SD (44.3%), and 27 cases of PD (9.1%); the ORR was 46.6% (139/298 cases) and DCR was 90.9% (271/298 cases). The median progression-free survival (mPFS) was 14.5 months (95% CI: 11.9-17.1), and the 1-, 2- and 3-year progression-free survival rates were 53.7%, 33.2%, and 24.6%, respectively; median overall survival (mOS) was 36.5 months (95% CI: NE-NE), and the 1-, 2- and 3-year overall survival rates were 81.9%, 61.6% and 51.9%, respectively. Treatment characteristics are presented in **Table 3**; **Figure 1**.

**Table 3:** Progression-free survival rates and overall survival rates.

Time point	Progression-free survival rates(%)	Overall survival rates(%)
1-year	53.7	81.9
2-year	33.2	61.6
3-year	24.6	51.9

### 3.3 Univariate analysis and multivariate analysis

The univariate analysis indicated that advanced age (≥65 years) (HR 1.64, 95% CI: 1.13-2.40, *P* = 0.010), worse physical strength score (PS≥1) (HR 1.83, 95% CI: 1.17-2.86, *P* = 0.008) and number of metastatic organs >2 (HR 1.72, 95% CI: 1.04-2.86, *P* = 0.036) were significantly associated with worse OS outcomes (**Table 4**). Multivariate analysis showed that no factors reached a statistical significance level of P < 0.05. Given the biological inclusion relationship between specific organ metastasis and the total number of metastatic organs, multivariate Cox regression models were constructed separately for subsequent analysis. The results revealed that, after correcting for other confounding factors, bone metastasis (HR 1.60, 95% CI: 1.02-2.50, *P* = 0.042) and the number of metastatic organs (HR 1.76, 95% CI: 1.05-2.92, *P* = 0.031) were both independent predictors of prognosis (**Table 4**).

**Table 4:** Univariate analysis of prognostic factors for OS.

Characteristic		n (%)	Univariate analysis		Multivariate analysis	
			HR (95%CI)	*P*	HR (95%CI)	*P*
Sex	Female	45 (15.1)	-^*^	0.435		
	Male	253 (84.9)	1.24 (0.72-2.15)			
Age	<65	165 (55.4)	–	0.010	–	0.090
	≥65	133 (44.6)	1.64 (1.13-2.40)		1.42 (0.95-2.12)	
ECOG PS	0	110 (36.9)	–	0.008	–	0.119
	≥1	188 (63.1)	1.83 (1.17-2.86)		1.51 (0.90-2.55)	
Pathology	SCC^1^	147 (49.3)	–	0.922		
	non-SCC^2^	151 (50.7)	1.02 (0.70-1.49)			
Smoking	No	104 (34.9)	–	0.092	–	0.096
	Yes	194 (65.1)	1.41 (0.96-2.08)		1.43 (0.94-2.18)	
Previous chronic history	No	110 (36.9)	–	0.081	–	0.813
	Yes	188 (63.1)	1.44 (0.96-2.15)		1.06 (0.67-1.67)	
Brain metastasis	No	255 (85.6)	–	0.809		
	Yes	43 (14.4)	0.94 (0.55-1.60)			
Bone metastasis	No	234 (78.5)	–	0.061	–	0.042
	Yes	64 (21.5)	1.50 (0.98-2.30)		1.60 (1.02-2.50)	
Pulmonary metastasis	No	241 (80.9)	–	0.529		
	Yes	57 (19.1)	1.16 (0.74-1.82)			
Pleural metastasis	No	249 (83.6)	–	0.285		
	Yes	49 (16.4)	1.30 (0.81-2.09)			
Liver metastasis	No	271 (90.9)	–	0.095	–	0.220
	Yes	27 (9.1)	1.64 (0.81-3.32)		1.46 (0.80-2.67)	
Adrenal metastasis	No	277 (93.0)	–	0.147		
	Yes	21 (7.0)	1.62 (0.85-3.11)			
Number of metastatic organs	≤2	263 (88.3)	–	0.036	–	0.031^3^
	>2	35 (11.7)	1.72 (1.04-2.86)		1.76 (1.05-2.92)	
PD-L1 status^4^	<1%	33 (23.9)	-	0.973		
	1%-49%	64 (21.5)	1.05 (0.51-2.17)			
	≥50%	41 (29.7)	0.98 (0.45-2.12)			
First-line clinical stage	II-III	131 (44.0)	–	0.555		
	IV	167 (56.0)	1.12 (0.77-1.65)			
Radiotherapy history	No	233 (78.2)	–	0.333		
	Yes	65 (21.8)	0.79 (0.48-1.28)			

^*^Reference. ^1^SCC, squamous cell carcinoma. ^2^non-SCC, non-squamous cell carcinoma, included adenocarcinoma, adenosquamous carcinoma, adenocarcinoma with sarcomatosis, and undifferentiated carcinoma. ^3^Multivariate analysis of the number of metastatic organs in relation to age, ECOG PS, smoking status, and previous chronic history showed that only the number of metastatic organs was statistically significant. ^4^In the PD-L1 status subgroup, univariate analysis was performed using only 138 cases with complete PD-L1 data.

The univariate analysis for PFS indicated that bone metastasis (HR 1.41, 95% CI: 1.01-1.97, P = 0.044), lung metastasis (HR 1.46, 95% CI: 1.04-2.05, *P* = 0.031), liver metastasis (HR 1.69, 95% CI: 1.08-2.64, *P* = 0.021), and number of metastatic organs >2 (HR 1.76, 95% CI: 1.18-2.61, *P* = 0.005) were significantly associated with worse PFS outcomes. Similar to the results of the multivariate analysis for OS, we conducted multivariate COX regression analyses for PFS separately for specific organ metastasis and the number of metastatic organs. The results showed that the first-line clinical stage (HR 1.44, 95% CI: 1.05-1.97, *P* = 0.024) was an independent prognostic risk factor affecting patients’ PFS. Details are shown in **Table 5**.

**Table 5:** Prognostic factors for PFS (univariate analysis and multivariate analysis).

Characteristic		n (%)	Univariate analysis		Multivariate analysis	
			HR (95%CI)	*P*	HR (95%CI)	*P*
Sex	Female	45 (15.1)	-^*^	0.945		
	Male	253 (84.9)	1.01 (0.68-1.52)			
Age	<65	165 (55.4)	–	0.797		
	≥65	133 (44.6)	1.04 (0.78-1.39)			
ECOG PS	0	110 (36.9)	–	0.516		
	≥1	188 (63.1)	1.11 (0.82-1.50)			
Histology	SCC^1^	147 (49.3)	–	0.262		
	non-SCC^2^	151 (50.7)	1.18 (0.88-1.57)			
Smoking	No	104 (34.9)	–	0.952		
	Yes	194 (65.1)	1.01 (0.75-1.36)			
Previous chronic history	No	110 (36.9)	–	0.710		
	Yes	188 (63.1)	1.06 (0.79-1.43)			
Brain metastasis	No	255 (85.6)	–	0.988		
	Yes	43 (14.4)	1.00 (0.67-1.49)			
Bone metastasis	No	234 (78.5)	–	0.044	–	0.542
	Yes	64 (21.5)	1.41 (1.01-1.97)		1.12 (0.78-1.63)	
Pulmonary metastasis	No	241 (80.9)	–	0.031	–	0.399
	Yes	57 (19.1)	1.46 (1.04-2.05)		1.18 (0.81-1.71)	
Pleural metastasis	No	249 (83.6)	–	0.102		
	Yes	49 (16.4)	1.36 (0.94-1.95)			
Liver metastasis	No	271 (90.9)	–	0.021	–	0.172
	Yes	27 (9.1)	1.69 (1.08-2.64)		1.38 (0.87-2.21)	
Adrenal metastasis	No	277 (93.0)	–	0.353		
	Yes	21 (7.0)	1.28 (0.76-2.18)			
Number of metastatic organs	≤2	263 (88.3)	–	0.005	–	0.068
	>2	35 (11.7)	1.76 (1.18-2.61)		1.48 (0.97-2.25)	
PD-L1 status^3^	<1%	33 (23.9)	-	0.135		
	1%-49%	64 (21.5)	0.81 (0.48-1.36)			
	≥50%	41 (29.7)	0.57 (0.31-1.00)			
First-line clinical stage	II-III	131 (44.0)	–	0.003	–	0.024^4^
	IV	167 (56.0)	1.56 (1.16-2.10)		1.44 (1.05-1.97)	
Radiotherapy history	No	233 (78.2)	–	0.799		
	Yes	65 (21.8)	0.96 (0.68-1.35)			

^*^Reference. ^1^SCC, squamous cell carcinoma. ^2^non-SCC, non-squamous cell carcinoma, included adenocarcinoma, adenosquamous carcinoma, adenocarcinoma with sarcomatosis, and undifferentiated carcinoma. ^3^In the PD-L1 status subgroup, univariate analysis was performed using only 138 cases with complete PD-L1 data. ^4^Multivariate analysis of the number of metastatic organs and first-line clinical stage showed that first-line clinical stage was statistically significant.

Prior to multivariable Cox regression analysis, the collinearity diagnostic revealed that the VIF values for all variables included in the multivariable analysis ranged from 1.08 to 1.75, which are well below the threshold of 5, indicating that there are no significant multicollinearity issues in the model (**Supplementary Table 2**). Therefore, all variables were retained in the final model. The PH assumption was formally assessed for all candidate variables using the Schoenfeld residual test. As detailed in **Supplementary Table 3**, none of the variables demonstrated a statistically significant violation of the PH assumption for either OS or PFS (all P > 0.05).

### 3.4 Evaluation of the six prognostic scoring systems

**Figure 2:**
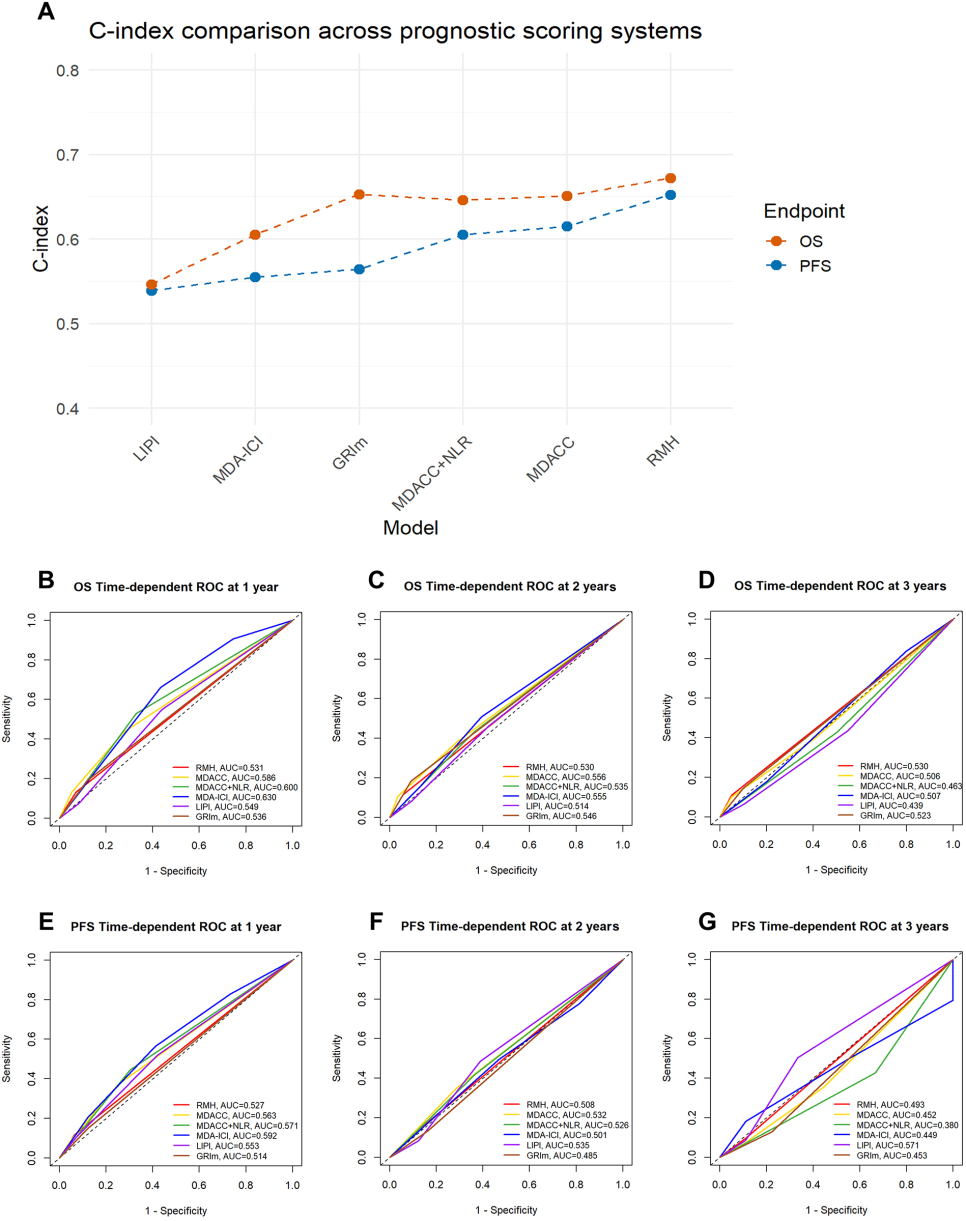
Comparison of performance across prognostic scoring systems in terms of c-index and time-dependent ROC curves. **(A)** The line chart compares the C-index values of six prognostic scoring systems—LIPI, MDA−ICI, GRIm, MDACC + NLR score, MDACC, and RMH—in predicting overall survival (OS) and progression-free survival (PFS). Higher C-index values indicate better discriminative ability of the model. **(B–G)** Time−dependent ROC curves for the 1−, 2−, and 3−year prediction of OS and PFS. The curves illustrate the discriminative performance of each prognostic scoring system over time, with corresponding AUC values annotated. AUC, Area Under Curve. The higher the AUC score, the better the model is able to classify observations into classes. A model with an AUC score of 0.5 is no better than a model that performs random guessing. 0.5 = No discrimination; 0.5-0.7 = Poor discrimination; 0.7-0.8 = Acceptable discrimination; 0.8-0.9 = Excellent discrimination; >0.9 = Outstanding discrimination.

To quantitatively compare predictive performance, we calculated the C-index and time-dependent AUC for each model. For OS, the RMH demonstrated the highest C-index of 0.672 (95% CI: 0.531-0.813), followed by the GRIm (C-index: 0.653, 95% CI: 0.538-0.768), MDACC (C-index: 0.651, 95% CI: 0.564-0.737) and MDACC + NLR (0.646, 95% CI: 0.554–0.738). In terms of 1-year AUC for OS, the MDA-ICI achieved the highest value of 0.630, whereas the MDACC + NLR showed a slightly higher 1-year AUC (0.600) compared to the original MDACC (0.586). However, the AUC values of all systems tended to decrease over time: at 3 years, the OS AUC ranged from 0.439 (LIPI) to 0.530 (RMH), with most systems showing values below 0.550. Regarding PFS, the RMH also exhibited the highest C-index of 0.652 (95% CI: 0.520-0.783). For the 1-year PFS AUC, the MDA-ICI again performed well with a value of 0.592. Similarly, the MDACC+NLR demonstrated a marginally higher 1-year AUC (0.571) than the MDACC (0.563). In general, the PFS AUC values of most systems also declined with prolonged follow-up. Detailed results are presented in **Table 6**; **Figure 2**.

**Table 6:** Comparison of predictive performance among prognostic scoring systems for PFS and OS.

Prognostic scoring system	OS				PFS			
	C-index (95% CI)	1-year AUC	2-year AUC	3-year AUC	C-index (95% CI)	1-year AUC	2-year AUC	3-year AUC
RMH	0.672 (0.531-0.813)	0.531	0.530	0.530	0.652 (0.520-0.783)	0.527	0.508	0.493
MDACC	0.651 (0.564-0.737)	0.586	0.556	0.506	0.615 (0.541-0.689)	0.563	0.532	0.452
MDACC + NLR	0.646 (0.554-0.738)	0.600	0.535	0.463	0.605 (0.531-0.679)	0.571	0.526	0.380
MDA-ICI	0.605 (0.535-0.676)	0.630	0.555	0.507	0.555 (0.498-0.611)	0.592	0.501	0.449
LIPI	0.546 (0.458-0.635)	0.549	0.514	0.439	0.539 (0.469-0.675)	0.553	0.535	0.571
GRIm	0.653 (0.538-0.768)	0.536	0.546	0.523	0.564 (0.453-0.684)	0.514	0.485	0.453

**Figure 3:**
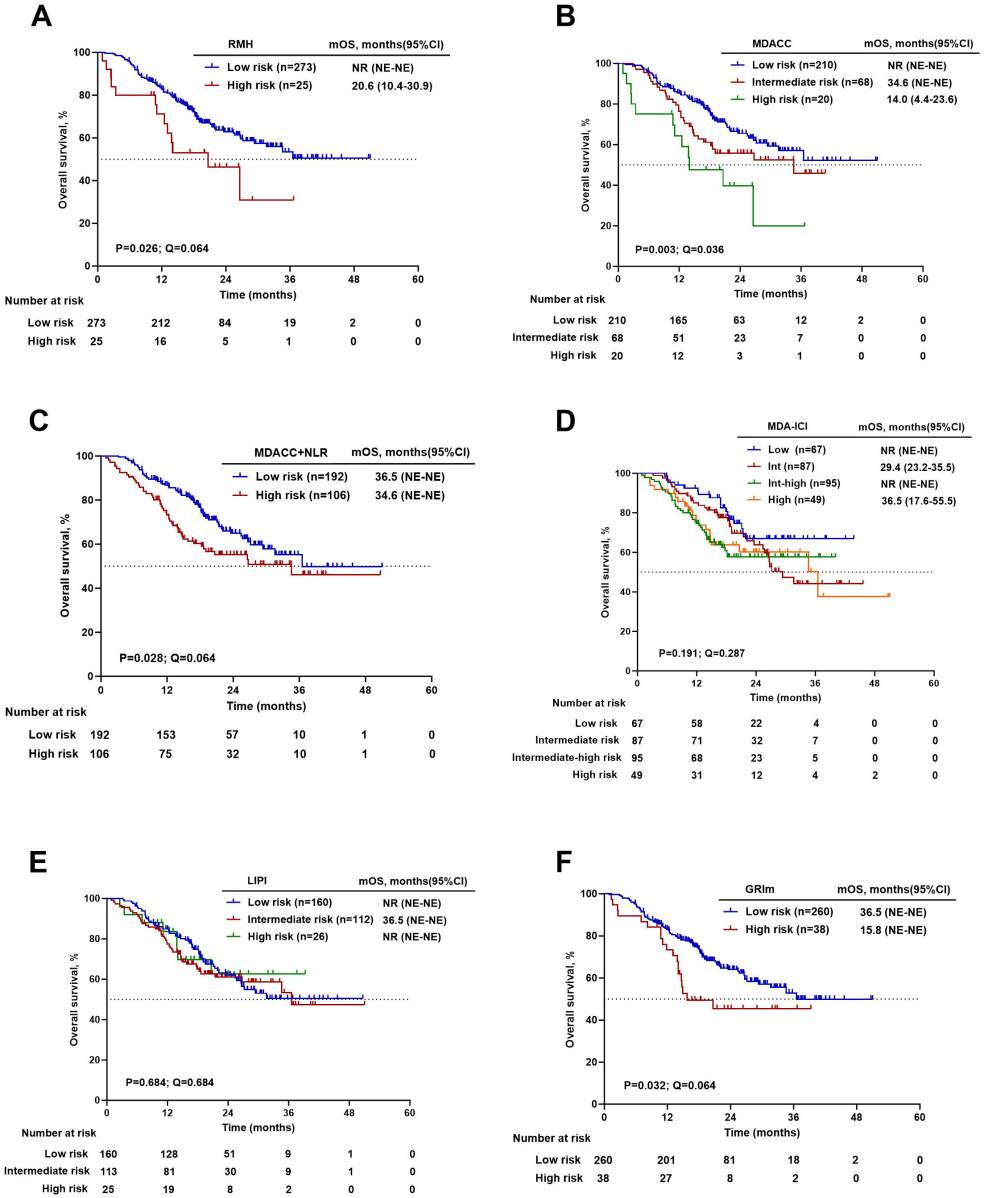
Kaplan - Meier survival curves for overall survival. **(A)** RMH high versus low risk. **(B)** MDACC high versus L versus low risk. **(C)** MDACC+NLR high versus low risk. **(D)** MDA-ICI high versus Intermediate-high versus intermediate versus low risk. **(E)** LIPI high versus intermediate versus low risk. **(F)** GRIm high versus low risk.

**Figure 4:**
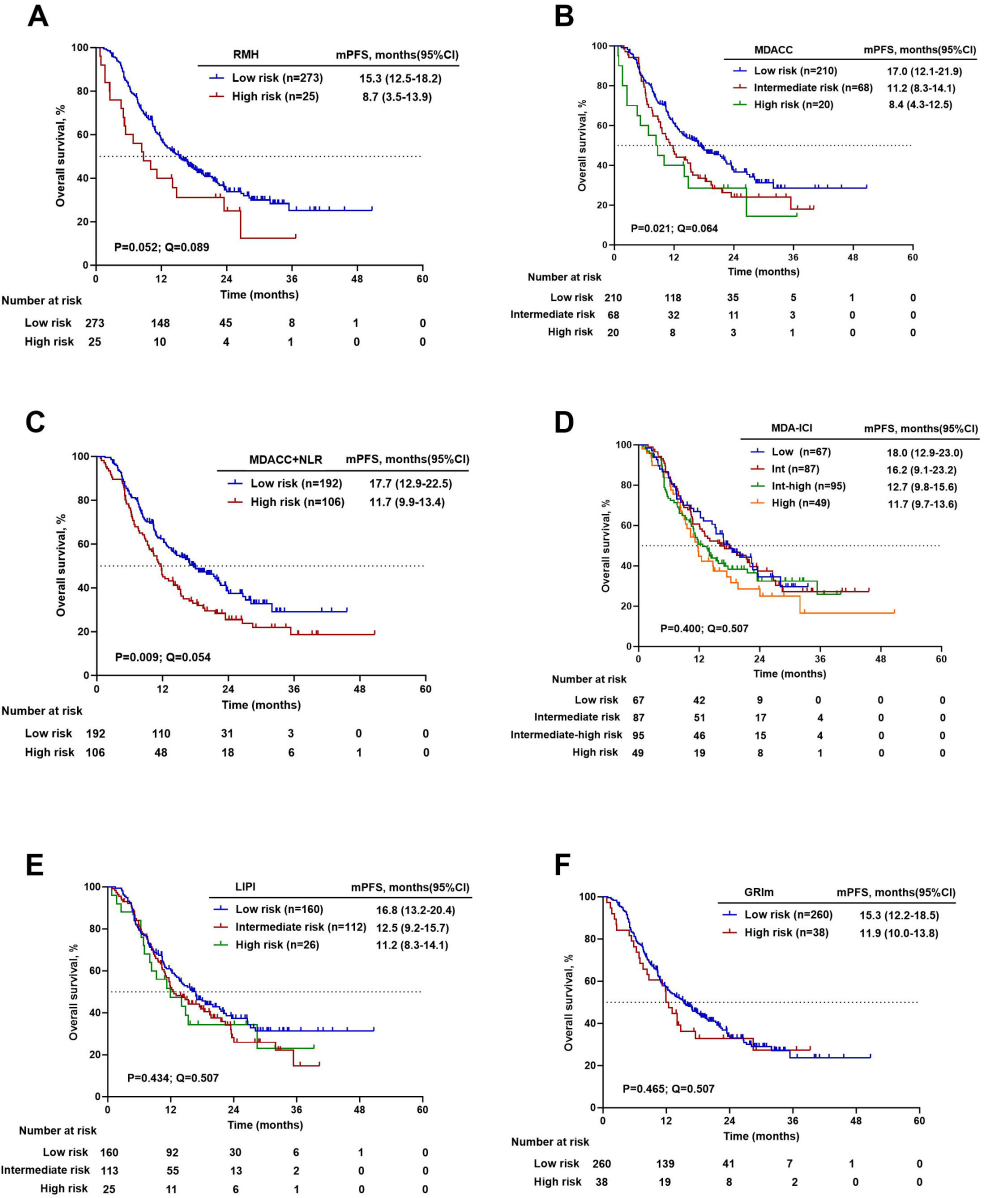
Kaplan–Meier survival curves for progression-free survival. **(A)** RMH high versus low risk. **(B)** MDACC high versus intermediate versus low risk. **(C)** MDACC+NLR high versus low risk. **(D)** MDA-ICI high versus Intermediate-high versus intermediate versus low risk. **(E)** LIPI high versus intermediate versus low risk. **(F)** GRIm high versus low risk.

Kaplan-Meier analysis with log-rank testing was performed to evaluate the prognostic stratification of the six scoring systems for both OS and PFS. The groups of risk categories are shown in **Table 1**. The RMH (20.6 vs. NR months, P = 0.026), MDACC (14.0 vs. 34.6 months vs. NR months, P = 0.003), MDACC+NLR (34.6 vs. 36.5 months, P = 0.028), and GRIm (15.8 vs. 36.5 months, P = 0.032) scores had significant differences in OS between the high-risk group and the low-risk group. For PFS, significant stratification was observed for the MDACC (8.4 vs. 11.2 vs. 17.0 months, P = 0.021) and MDACC+NLR (11.7 vs. 17.7 months, P = 0.009) scores, while the RMH score showed a borderline trend (8.7 vs. 15.3 months, P = 0.052). After adjustment for multiple comparisons using the FDR method, only the MDACC score remained significantly associated with OS (14.0 vs. 34.6 vs. NR months, P = 0.003, Q = 0.036). For PFS, while the MDACC+NLR score showed the strongest nominal association, its corrected Q value (0.054) marginally exceeded the significance threshold. The associations of other scores with either OS or PFS were not significant after FDR correction. Details are shown in **Table 7**. Kaplan–Meier estimates of OS and PFS stratified by prognostic risk category for each score are presented in **Figures 3**, **4**.

**Table 7:** Grouping and survival analysis for prognostic risk groups according to six scoring systems.

Prognostic scoring system		Number(%)	OS (months)			PFS(months)		
			mOS (95%CI)	*P*-value	*Q*-value	mPFS(95%CI)	*P*-value	*Q*-value
RMH	0-1	273 (91.6)	NR (NE-NE)	0.026	0.064	15.3 (12.5-18.2)	0.052	0.089
	2-3	25 (8.4)	20.6 (10.4-30.9)			8.7 (3.5-13.9)		
MDACC	0-1	210 (70.5)	NR (NE-NE)	0.003	0.036*	17.0 (12.1-21.9)	0.021	0.064
	2	68 (22.8)	34.6 (NE-NE)			11.2 (8.3-14.1)		
	3-5	20 (6.7)	14.0 (4.4-23.6)			8.4 (4.3-12.5)		
MDACC+NLR	0-1	192 (64.4)	36.5 (NE-NE)	0.028	0.064	17.7 (12.9-22.5)	0.009	0.054
	>1	106 (35.6)	34.6 (NE-NE)			11.7 (9.9-13.4)		
MDA-ICI	0-2	67 (22.5)	NR (NE-NE)	0.191	0.287	18.0 (12.9-23.0)	0.400	0.507
	3	87 (29.2)	29.4 (23.2-35.5)			16.2 (9.1-23.2)		
	4	95 (31.9)	NR (NE-NE)			12.7 (9.8-15.6)		
	5-7	49 (16.4)	36.5 (17.6-55.5)			11.7 (9.7-13.6)		
LIPI	0	160 (53.7)	NR (NE-NE)	0.684	0.684	16.8 (13.2-20.4)	0.434	0.507
	1	112 (37.6)	36.5 (NE-NE)			12.5 (9.2-15.7)		
	2	26 (8.7)	NR (NE-NE)			11.2 (8.3-14.1)		
GRIm	0-1	260 (87.2)	36.5 (NE-NE)	0.032	0.064	15.3 (12.2-18.5)	0.465	0.507
	2-3	38 (12.8)	15.8 (NE-NE)			11.9 (10.0-13.8)		

P values were derived from log-rank tests comparing survival across risk groups defined by each scoring system. To account for multiple comparisons across six different prognostic models, all P values were adjusted using the Benjamini-Hochberg method to control the false discovery rate (FDR). The resulting Q values are reported. A Q value < 0.05 was considered statistically significant after correction (*).

## 4 Discussion

NSCLC is a prevalent and aggressive form of cancer that exhibits a wide range of clinicopathological characteristics and has a high likelihood of metastasis (11). Traditional platinum-based chemotherapy has shown limited efficacy in treating patients with advanced NSCLC who do not harbor driver gene mutations (12). However, the emergence of immunotherapeutic agents, known as immune checkpoint inhibitors (ICIs), has revolutionized the treatment landscape and led to improved survival rates when used in combination with chemotherapy (13, 14). Despite these advancements, the overall prognosis for NSCLC patients remains unfavorable. Therefore, it is crucial to identify valuable prognostic factors that can help categorize patients into homogeneous subgroups based on their clinicopathological features. Constructing a prognostic scoring system based on these factors can provide guidance for clinicians in tailoring treatment strategies for individual patients.

Interim data from the CameL study showed that the combination of Camrelizumab and chemotherapy significantly improved mPFS compared to chemotherapy alone (11.3 vs. 8.3 months, HR = 0.60, *P* = 0.0001) (14). Similarly, the KEYNOTE-189 study demonstrated improved OS and PFS for patients receiving pembrolizumab plus platinum-based chemotherapy, with a mOS of 22.0 months and mPFS of 9.0 months (15). In the KEYNOTE-407 Chinese extension study, the pembrolizumab combination group had an mOS of 30.1 months and mPFS of 8.3 months, compared to 12.7 months and 4.2 months in the placebo group, respectively (16). The 1- and 2-year OS rates were 78.5% and 56.9%, and the 1- and 2-year PFS rates were 35.4% and 24.2% in the pembrolizumab group (16). Although the OS data from our study are still immature, a synthesis of previous evidence-based research indicates a promising trend of significant improvement in mOS and mPFS for driver gene-negative NSCLC patients receiving first-line CIT. These findings align with the conclusions drawn from previous studies.

Although VIF analysis did not indicate significant multicollinearity, specific organ metastasis and the number of metastatic organs may convey overlapping prognostic information. When included simultaneously in the same model, the shared variance between these variables may reduce their apparent independent effects, leading to attenuation of statistical significance. Therefore, we included the specific organ transfer status and the number of transferred organs separately in the final multivariate analysis. The results showed that bone metastasis and more than two metastatic organs were poor prognostic factors for OS, while first-line clinical stage was a risk factor for PFS. Prior research indicates that the percentages of NSCLC paired with single brain, bone, lung, liver, and multi-organ metastases were 15.4%, 22.3%, 20.1%, 6.1%, and 36.1%, respectively (17). Different baseline metastatic organs may affect the immunotherapy response differently. Previous studies have found that the number of distant metastatic organs is a prognostic factor in patients with advanced NSCLC (18, 19). The finding indicates that as the extent of metastasis increases, the risk of mortality rises significantly, which further support the crucial role of tumor burden in survival outcomes. Bauml et al. retrospectively found that baseline bone metastases was independent risk factor for shorter survival (20), which is generally consistent with the results of this study. Poor immunotherapy response has been observed in patients with NSCLC liver metastases, which is mostly associated with the immunosuppressive characteristics of the hepatic immune microenvironment (21). In contrast, liver metastasis did not reach statistical significance in the multivariate analysis in this study (P = 0.220). This may be due to the limited sample size or the fact that the impact of liver metastasis on overall prognosis was attenuated by other factors.

In immunotherapy, prognostic ratings have drawn more attention as a means of forecasting the survival benefit of patients. High expression of PD-L1 (tumor proportion score ≥50%) has been validated as a predictive biomarker for the efficacy of PD-1/PD-L1 inhibitor monotherapy across multiple clinical trials, including KEYNOTE-024 (22) and KEYNOTE-042 (23), indicating that patients with PD-L1 ≥50% derive greater benefit from single-agent immunotherapy. However, in the setting of immune checkpoint inhibitors combined with platinum-based chemotherapy—as demonstrated in phase III trials such as KEYNOTE-189 (24), IMPOWER132 (25), and GEMSTONE-302 (26)—significant improvements in OS and PFS have been observed regardless of PD-L1 expression levels. In the present study, PD-L1 testing was not routinely performed due to factors such as the lack of mandatory guidelines, limited tissue availability, and economic or time constraints, resulting in a high rate of missing PD-L1 expression data. Among the 298 enrolled patients, only 138 (46.3%) underwent PD-L1 testing. Subsequent stratified survival analysis based on PD-L1 TPS categories (<1%, 1–49%, and ≥50%) revealed no significant association between PD-L1 expression levels and treatment outcomes in our cohort (P > 0.1; **Tables 4**, **5**). Given these real-world limitations, this study aimed to explore a more readily available, cost-effective, and clinically accessible assessment tools. The RMH, MDACC, MDACC+NLR, MDA-ICI, LIPI, and GRIm scoring systems, which consist of more readily available clinicopathologic features and peripheral blood biomarker metrics, are applied in the context of clinical phase I trials, but the predictive value of first-line CIT in NSCLC patients’ needs to be further validated.

A direct comparative evaluation of discriminative performance reveals the core differences among the prognostic score systems. Overall, the RMH score appeared to show stronger discrimination for OS and PFS. However, its wide 95% CI indicates considerable uncertainty in this estimate, suggesting potentially unstable performance. The GRIm, MDACC, and MDACC + NLR scores achieved similar and moderate C-indices for OS. In terms of predicting PFS, both the MDACC score and the MDACC+NLR score are second only to the RMH criteria in predictive ability. More importantly, time-dependent AUC analysis uncovered temporal heterogeneity in the predictive efficacy of the models. The MDA-ICI and MDACC+NLR scores excelled in short-term (1-year) OS and PFS prediction, indicating its unique value in identifying patients at immediate high risk. A universal and critical finding was the notable decline in the 3-year AUC for all models, exposing a common limitation of existing static baseline scoring systems in predicting long-term outcomes. Long-term prognosis is likely influenced more by dynamic factors during treatment, such as subsequent therapies and tumor evolution.

After stringent adjustment for multiple comparisons, only the MDACC score emerged as the most robust predictor for OS in our cohort. This finding suggests that, despite not achieving the highest C-index, MDACC provides the most reliable and reproducible survival stratification in this cohort, with the lowest likelihood that observed differences occurred by chance. Conversely, while the RMH score led in discriminative metrics, its Q-values for both OS and PFS did not reach the significance threshold. The MDACC+NLR score showed promise for PFS prediction, where its raw P-value (0.009) and borderline Q-value (0.054).

Based on the integrated evaluation of discriminative performance and statistical robustness, our findings suggest that these prognostic scoring systems should be applied in a context‐dependent manner. When the highest degree of statistical rigor and inferential certainty for OS is paramount, the MDACC score is recommended. This is particularly relevant in contexts such as confirmatory research or pivotal clinical decision-making, as it was the sole model retaining a significant association with OS after strict multiple testing correction. In contrast, the RMH score achieved the highest C-indices for both OS and PFS, indicating superior discriminative ability; however, the limited statistical robustness of its survival stratification and the small proportion of high-risk patients warrant cautious interpretation, particularly when drawing definitive clinical conclusions. The MDACC+NLR score showed particular strength in short-term outcome prediction, with favorable 1-year AUCs for both OS and PFS and a borderline significant association with PFS after multiple testing adjustment, suggesting that this model may be especially useful in clinical scenarios or trial designs focused on early events such as rapid disease progression. Notably, the time-dependent AUCs of all models declined at later time points. This pattern reflects the increasing influence of post-progression treatments, disease evolution, and unmeasured clinical or biological factors on long-term survival outcomes in real-world settings. These findings highlight the intrinsic limitations of static baseline prognostic scores in predicting long-term survival for patients with advanced NSCLC receiving first-line chemo-immunotherapy. Importantly, the aim of this study was not to provide precise individual-level survival prediction, but to perform a head-to-head comparison of widely used prognostic scoring systems under identical clinical conditions. Within this context, relative differences in model performance remain informative for risk stratification and population-level prognostic assessment, despite the modest absolute discrimination.

MDA-ICI, LIPI, and GRIm scores did not demonstrate consistent or robust prognostic performance in this cohort, limiting their utility as standalone tools. The low reproducibility of the MDA-ICI system could be due to the dichotomization of continuous variables to determine thresholds, which is challenging for peripheral blood indicators that exhibit significant fluctuations. Moreover, the original MDA-ICI and GRIm data included advanced cancer patients on ICI monotherapy, unlike the cohort of CIT patients in this study, suggesting the need for further research to assess their predictive utility in NSCLC patients receiving first-line CIT. Regarding LIPI, previous studies have reported heterogeneous results. In some cohorts, patients in the high-risk LIPI group did not demonstrate a significant OS benefit compared with those receiving chemotherapy alone (27). Conversely, a study of advanced NSCLC patients treated with nivolumab monotherapy reported that a poor LIPI score was associated with shorter OS (HR 3.67, 95% CI: 1.96–6.86, P < 0.0001), but not with PFS (HR 1.49, 95% CI: 0.94–2.38, P = 0.090) (28). Hopkins et al. found significantly prolonged OS (mOS: 24 *vs.* 16 *vs.* 7 months, *P* < 0.001) in the low-risk group of LIPI compared to the high-risk group in patients with NSCLC treated with atalizumab in combination with chemotherapy and bevacizumab regimens (29). The disappointing results in this study may be due to differences in treatment regimens and patient characteristics.

Indeed, most statistical models are derived from data-driven sources, leading to possible biases in prognostic scoring model development, assignment of scores, and performance of the resulting risk groups (30). The reliability and generalizability of prognostic scoring systems is precisely established through efficacy testing in diverse settings (31). In this study, we hope to assess the predictive value of the current six prognostic scores in a cohort of NSCLC patients receiving first-line CIT combinations with external data validation, to inform the selection and improvement of prognostic scoring models for subsequent clinical practice, and to help clinicians to make more accurate decisions about the development of immunotherapy regimens and prediction of prognosis for this group of patients.

This study also has many shortcomings. First, this study was a bidirectional cohort study with a total of 298 patients enrolled, all of whom were patients with recurrent or metastatic NSCLC treated with first-line CIT included from a single center, with a limited sample size, which may have a certain selective bias; second, from this study to the final follow-up date of December 31, 2023, a total of 109 cumulative deaths (36.5%) were recorded, and some of the patients were still under treatment, which somewhat affected the acquisition of survival data such as mOS. Due to the limited number of OS events, the results—particularly in smaller subgroups—must be interpreted with caution as they are preliminary and unstable. Third, this study included mainly driver gene-negative NSCLC patients, and due to the different panel of genetic testing methods used by the patients, eight genes, including EGFR, ALK, ROS1, RET, HER-2, c-MET, BRAF, and NTRK, were mainly excluded, and the status of the other concomitant genes was not completely clear. Fourth, the high missing rate of PD-L1 data in this study, due to practical constraints, may limit the interpretation and generalizability of our findings. Fifth, patients in this study received a diverse array of therapeutic agents. This variation in treatment regimens represents a potential confounding factor that may limit comparability.

While this study provides initial insights under real-world constraints, the limitations highlight critical goals for subsequent work. Advancing this field will require prospective, multi-center validation in broader populations, coupled with standardized molecular profiling and systematic biomarker collection. Such efforts are key to translating prognostic tools into reliable clinical applications.

## Data Availability

The datasets used and/or analyzed during the current study are available from the corresponding author on reasonable request. All the data is stored by the Fourth Hospital of Hebei Medical University. To protect the privacy of study participants, the data involved in this study is not publicly shared.

## Supplementary material

Supplementary Table 1 Incidence of TRAEs in NSCLC patients receiving first-line CIT. The collection of adverse events in this study relied solely on medical record documentation. Due to the inherent limitations in the completeness of medical records, some adverse events may have been underreported. Therefore, the incidence rates of adverse events reported in this study may not fully reflect the actual clinical incidence.

Supplementary Table 2 Variance inflation factors for variables in the Cox proportional hazards models of OS and PFS. *VIF, Variance Inflation Factor.

Supplementary Table 3 Schoenfeld residual tests for the proportional hazards assumption in the multivariate Cox model of OS and PFS.
