# Supplementary material for: Evaluation of six clinical prognostic scores in NSCLC patients undergoing first line chemoimmunotherapy
Source: Front Immunol. 2026 Feb 17;17:1695859. doi: 10.3389/fimmu.2026.1695859 (PMC12953529; doi:10.3389/fimmu.2026.1695859)
Supplement: Supplementary Table 1 — Incidence of TRAEs in NSCLC patients receiving first-line CIT. The collection of adverse events in this study relied solely on medical record documentation. Due to the inherent limitations in the completeness of medical records, some adverse events may have been underreported. Therefore, the incidence rates of adverse events reported in this study may not fully reflect the actual clinical incidence. [file Table1.docx]

| **TRAEs^1^** | **Grade1-2, n (%)** | **Grade 3-4, n (%)** | **Total, n (%)** |
| --- | --- | --- | --- |
| **All TRAEs** | 175 (58.7%) | 113 (37.9%) | 288 (96.6%) |
| **Hematologic adverse events** |  |  |  |
| **Leukopenia** | 101 (33.9%) | 69 (23.2%) | 139 (46.6%) |
| **Neutropenia** | 93 (31.2%) | 68 (22.8%) | 125 (41.9%) |
| **Thrombocytopenia** | 56 (18.8%) | 27 (9.1%) | 83 (27.9%) |
| **Anemia** | 164 (55.0%) | 38 (12.8%) | 202 (67.8%) |
| **Gastrointestinal adverse events** |  |  |  |
| **Anorexia** | 127 (42.6%) | 14 (4.8%) | 141 (47.3%) |
| **Nausea** | 108 (36.2%) | 14 (4.8%) | 122 (40.9%) |
| **Vomiting** | 49 (16.4%) | 14 (4.8%) | 110 (36.9%) |
| **Elevated ALT^2^/AST^3^** | 50 (16.9%) | 13 (4.2%) | 63 (21.1%) |
| **Elevated bilirubin** | 19 (6.8%) | 4 (1.3%) | 23 (7.7%) |
| **Urinary system adverse events** |  |  |  |
| **Elevated serum creatinine** | 14 (4.7%) | 0 (0%) | 14 (4.7%) |
| **Fatigue** | 112 (37.6%) | 18 (6.0%) | 130 (43.6%) |
| **Fever** | 27 (9.1%) | 3 (1.0%) | 30 (10.1%) |
| **All irAEs^4^** | 82 (27.5%) | 15 (5.0%) | 87 (33.9%) |
| **Immune-related skin disorders** | 28 (9.4%) | 3 (1.0%) | 31 (10.4%) |
| **Immune-related diarrhea/colitis** | 21 (7.1%) | 2 (0.7%) | 23 (7.7%) |
| **Immune-related pneumonitis** | 24 (8.1%) | 9 (3.0%) | 33 (11.1%) |
| **Immune-related hypothyroidism** | 13 (4.4%) | 0 (0%) | 13 (4.4%) |
| **Immune-related hypopituitarism** | 3 (1.0%) | 0 (0%) | 3 (1.0%) |
| **Immune-related adrenal insufficiency** | 5 (1.7%) | 1 (0%) | 6 (2.0%) |

**Supplementary Tab.1 Incidence of TRAEs in NSCLC Patients Receiving First-Line CIT.**

The collection of adverse events in this study relied solely on medical record documentation. Due to the inherent limitations in the completeness of medical records, some adverse events may have been underreported. Therefore, the incidence rates of adverse events reported in this study may not fully reflect the actual clinical incidence.

^1^TRAEs, treatment-related adverse events. ^2^ALT, alanine transaminase. ^3^AST, aspartate transaminase. ^4^irAEs, immune-related adverse events.

| Characteristics for OS | VIF* | Characteristics for PFS | VIF |
| --- | --- | --- | --- |
| Age | 1.16 | Bone metastesis | 1.47 |
| ECOG PS | 1.49 | Pulmonary metastesis | 1.39 |
| Smoking | 1.08 | Liver metastesis | 1.38 |
| Previous chronic history | 1.41 | Number of metastatic sites | 1.75 |
| Bone metastesis | 1.30 | First-line clinical stage | 1.53 |
| Liver metastesis | 1.37 | - | - |
| Number of metastatic sites | 1.54 | - | - |

**Supplementary Tab.2** **Variance Inflation Factors (VIFs) for Variables in the Cox Proportional Hazards Models of OS and PFS.**

*VIF, Variance Inflation Factor.

| **Variable** | **OS** | | |  | **PFS** | | |
| --- | --- | --- | --- | --- | --- | --- | --- |
|  | **χ²** | **df** | **P-value** |  | **χ²** | **df** | **P-value** |
| **Age (years)** | 2.614 | 1 | 0.106 |  | - | - | - |
| **ECOG PS** | 1.321 | 1 | 0.250 |  | - | - | - |
| **Smoking** | 1.843 | 1 | 0.175 |  | - | - | - |
| **Previous chronic history** | 2.871 | 1 | 0.090 |  | - | - | - |
| **Bone metastesis** | 0.104 | 1 | 0.747 |  | 0.642 | 1 | 0.423 |
| **Pulmonary metastesis** | - | - | - |  | 0.698 | 1 | 0.404 |
| **Liver metastesis** | 0.011 | 1 | 0.917 |  | <0.001 | 1 | 0.985 |
| **Number of metastatic sites** | 0.092 | 1 | 0.762 |  | 0.109 | 1 | 0.741 |
| **First-line clinical stage** | - | - | - |  | 0.213 | 1 | 0.645 |
| **GLOBAL** | 12.360 | 7 | 0.089 |  | 2.306 | 5 | 0.805 |

**Supplementary Tab.3 Schoenfeld residual tests for the proportional hazards assumption in the multivariate Cox model of OS and PFS.**
